# Performance Analysis of Electromyogram Signal Compression Sampling in a Wireless Body Area Network

**DOI:** 10.3390/mi13101748

**Published:** 2022-10-15

**Authors:** Liangyu Zhang, Junxin Chen, Chenfei Ma, Xiufang Liu, Lisheng Xu

**Affiliations:** 1 College of Medicine and Biological Information Engineering, Northeastern University, 195 Innovation Road, Shenyang 110169, China; 2 Edinburgh Neuroprosthetics Laboratory, School of Informatics, The University of Edinburgh, 10 Crichton Street, Edinburgh EH8 9AB, UK; 3 Paul C. Lauterbur Research Center for Biomedical Imaging, Shenzhen Institutes of Advanced Technology, Chinese Academy of Sciences, Shenzhen 518055, China

**Keywords:** compressed sensing, electromyogram, reconstruction algorithm, wavelet basis

## Abstract

The rapid growth in demand for portable and intelligent hardware has caused tremendous pressure on signal sampling, transfer, and storage resources. As an emerging signal acquisition technology, compressed sensing (CS) has promising application prospects in low-cost wireless sensor networks. To achieve reduced energy consumption and maintain a longer acquisition duration for high sample rate electromyogram (EMG) signals, this paper comprehensively analyzes the compressed sensing method using EMG. A fair comparison is carried out on the performances of 52 ordinary wavelet sparse bases and five widely applied reconstruction algorithms at different compression levels. The experimental results show that the db2 wavelet basis can sparse EMG signals so that the compressed EMG signals are reconstructed properly, thanks to its low percentage root mean square distortion (PRD) values at most compression ratios. In addition, the basis pursuit (BP) reconstruction algorithm can provide a more efficient reconstruction process and better reconstruction performance by comparison. The experiment records and comparative analysis screen out the suitable sparse bases and reconstruction algorithms for EMG signals, acting as prior experiments for further practical applications and also a benchmark for future academic research.

## 1. Introduction

Electromyography (EMG) is a technique for evaluating and recording the electrical activity produced by skeletal muscles [1]. Electromyography is performed using an instrument called an “electromyograph” to generate a record of electromyography. When myocytes are activated by electricity or nerves, electromyography can detect the potential that is generated. The signals can be analyzed to detect medical abnormalities, activation levels or recruitment sequences, or to analyze the biomechanics of human or animal movements. In computer science, EMG is also used as a middleware for gesture recognition to allow physical actions to be input into a computer as a form of human computer interaction [2]. The efficient acquisition, storage and transmission of electromyography (EMG) data is important for emerging applications such as telemedicine. However, the transmission and storage of EMG data is challenging due to limitations relating to Internet speed and hardware resources. These problems have been effectively solved with the emergence of wireless body sensor networks (WBSNs) [3,4,5].

Compressed sensing is widely implemented in the WBSN to reduce the power consumption of the sensors and to increase the security of the data. Xu et al. presented a compressed sensing-based approach to co-recognize human activities and sensor locations in a single framework [6]. Kimia et al. proposed a new method for EMG data compression using deep convolutional autoencoders (CAE) [7]. Shoaib et al. proposed a method to capture and detect EEG signals based on compressed perception, which reduced the energy consumption during communication and computation [8]. Zhang et al. came up with a method called block sparse Bayesian learning (BSBL) to solve the problem of compressed sensing and applied it for long-distance EEG monitoring [9]. Imtiaz et al. proposed a low-power MSP430 compressive sensing implementation, focusing mainly on the impact of the sensor node architecture on the compression performance [10]. Liu et al. solved the problem whereby EEG signals cannot be represented sparsely and shortened the computing time of single-channel EEG signals in compressed sampling. An optimization model with the $\;l_{0}$ norm and Schatten-0 norm was proposed to enforce sparsity and low-rank structures in the reconstructed multi-channel EEG signals [11]. Selin Aviyente applied a compression sensing framework for EEG signal compression [12], and the Gauss random matrix was utilized to compress the input signal in the measurement. In the reconstruction process, a discrete cosine matrix was selected as a sparse basis and the EEG signal recovery was completed by combining it with an orthogonal matching pursuit (OMP) algorithm [13]. Amir et al. [14] proposed a reconstruction scheme analysis framework, which employed a Gaussian random matrix to compress the original signal. Then, different sparse bases and several main reconstruction algorithms were combined separately. After this, they exploited the compressive sensing theory to analyze the refactored EEG signals and analyzed the performances of various combinations in detail. Zhao et al. [15] studied and improved the disadvantages of the high power consumption and large area of random dense binary measurement matrices. They proposed the QCAC matrix and random sparse binary matrix, and achieved a positive signal reconstruction effect while saving hardware computing resources. Moreover, they implemented the two measurement matrices in hardware based on a 65 nm CMOS processor. Emil Jovanov and Aleksandar Milenkovic designed the infrastructure for a wireless body area network based on an intelligent motion sensor for computer-aided physical rehabilitation [5]. In 2010, Hyunwoong Park put forward a space–time block coding (STBC) scheme based on a double transmitter and single receiver. They studied the influence of three communication channels on the body, through the body, diffraction around the body, and reflection of the body, and composed a propagation channel model of the body’s surface [16].

Mamaghanian et al. applied the hardware platform of a shimmer wireless body area network to compare the compression performance for ECG based on a wavelet transform and compressed sensing. They indicated that the compressed sensing of ECG signals consumed less power and was suitable for the body area network with high real-time requirements [17]. Zhang et al. exploited the intra-block correlation of ECG signals under the framework of the compressed sensing theory and came up with a reconstruction algorithm based on block sparse Bayesian learning. Compared with other reconstruction algorithms, this algorithm has better ECG reconstruction performance and was beneficial in the data analysis and diagnosis of body area network telemonitoring centers [18]. Dixon et al. presented a 1-bit Bernoulli compressed sensing observation matrix for the dynamic threshold method of ECG signals, which reduced the transmission power consumption of body area network sensor nodes by increasing the compression ratio [19].

In this paper, we applied EMG compressed sensing on wireless body area networks. A fixed measurement matrix and reconstruction algorithm were selected and the most suitable wavelet base for the EMG signal was determined according to the reconstruction quality of the EMG signal. Our research was based on the existing research results, adding different sparse bases and reconstruction algorithms and evaluating the compression sensing performance of the EMG signals. The contributions can be summarized as follows: (1) The fixed measurement matrix (Bernoulli matrix) and the fixed BP reconstruction algorithm were employed [20]. Fifty-two kinds of sparse wavelet bases were selected, and the compression ratio range was set from 10% to 90%. Finally, after sparse decomposition and reconstruction, the average PRD value for 500 segments of EMG signals for each wavelet basis was calculated. From this, the most suitable wavelet basis for the EMG signals was evaluated. (2) A fixed measurement matrix (Bernoulli matrix) and wavelet basis were applied. Five reconstruction algorithms were evaluated via PRD, along with the time consumed. (3) The future development prospects were discussed and trends were identified.

The remainder of this paper is structured as follows. The basic concepts of CS and its application in WBSN are elucidated in the Methods section. In the results section, the compressed sampling results based on the EMG signals are shown and compared in detail. Finally, in the discussion section, the experimental results are summarized, the deficiencies are put forward, and the future implementation plan is preliminarily formulated.

## 2. Materials and Methods

### 2.1. WBSN

As a communication network for the long-distance, real-time continuous monitoring of a patient’s physical conditions, important links applied via the Internet of Things (IoT) [21,22]. A WBSN is a wireless network for wearable devices [23,24,25,26]. It transmits physiological signal data between patients and hospitals far away via wireless networks based on wearable devices [27]. As a portable device, it has intimate connections with the surface of the human skin [28]. Although the acquisition device is small, the application management system in the data center and the user interface require a larger intelligent device and greater support [29]. WBSNs became popular at the end of the 20th century. The original idea was to connect individuals and surrounding devices based on a wireless personal area network (WPAN). To cover a more expansive space, WPAN technology is utilized as the gateway of the WBSN system so that the device placed in the human body can be connected to the Internet. Benefiting from this, medical staff can read the physiological data of remote patients in real time through the Internet.

**Figure 1:**
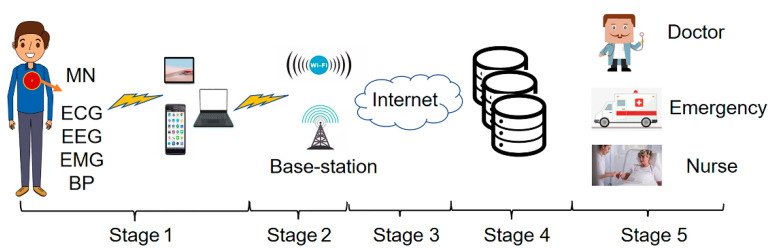
Real-time monitoring and remote transmission of human physiological signals.

As shown in Figure 1, there are five stages for the real-time monitoring and remote transmission of human physiological signals.

Stage1: Physiological signal acquisition stage. Sensors for measuring ECG, EEG, EMG, and other physiological signals are placed on the body’s surface or implanted under the skin. The data collected by the sensors are sent to the master node (MN) following IEEE standard 802.15.6. Then, the MN transmits the data to the local mobile devices (mobile phones, tablets, laptops) through wireless media and the local processing unit transmits the data to the next level.

Stage2: Wireless communication transmission stage. The further transmission of physiological signal data is achieved through the access point, WIFI, or cellular base station.

Stage3: Internet transmission stage. In this stage, long-distance data transmission is achieved through optical fiber technology.

Stage4: Data storage and analysis stage. The physiological signal data transmitted by long-distance are stored and analyzed in this stage. After the analysis, the data are classified and stored.

Stage5: Medical diagnosis and treatment stage. In this stage, the stored data are transmitted to the medical and health institutions. The doctor diagnoses the patient’s condition according to the remote output data and gives the treatment plan [30].

Patient medical problems have caused various countries to bear many economic expenditures in recent years. WBSNs can be employed as a feasible solution to reduce medical expenses.

### 2.2. Compressed Sensing

**Figure 2:**
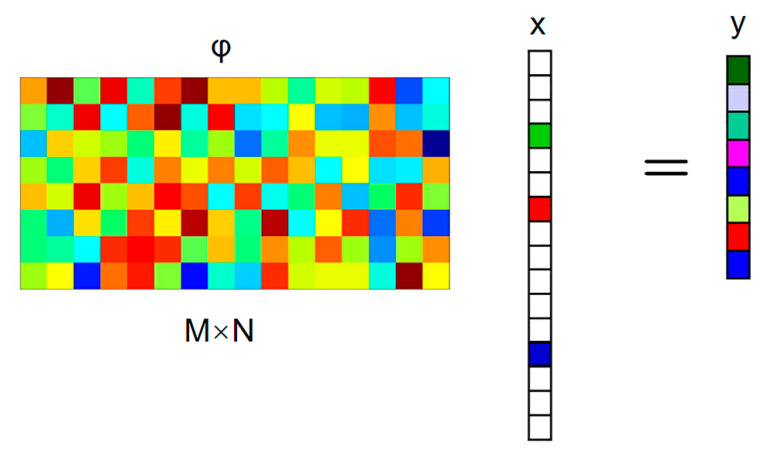
The CS sampling process.

Figure 2 shows the CS sampling process, in which the signal x to be collected is only non-zero at k times (k is the sparse degree). To manage the information in x, it is projected on a given set of sensing waveforms ϕ (that is to say, x is scented with a given set of waveforms). The most common measurement matrices are the Gaussian random matrix and the Bernoulli matrix, which do not correlate with the sparsest matrices. In addition, the measurement matrix can also use deterministic matrices such as a polynomial matrix, chaos matrix, and structured random matrix (e.g., the Toeplitz matrix).

The Bernoulli matrix is selected as a fixed measurement matrix in this paper. It constructs a matrix ϕ of size *M* × *N* in order that every element in ϕ independently obeys the Bernoulli distribution:

(1) $$\Phi_{i,j}=\{\begin{array}{cc} +\frac{1}{\sqrt{M}} & P=\frac{1}{2}\\ -\frac{1}{\sqrt{M}} & P=\frac{1}{2} \end{array}=\frac{1}{\sqrt{M}}\{\begin{array}{cc} +1 & P=\frac{1}{2}\\ -1 & P=\frac{1}{2} \end{array}$$

and a set of measures y, which is far less than the original length of the signal that is obtained:

(2) y=ϕx

When compressible, the method can also estimate the items with a larger amplitude. In practical applications, the signal *x* to be collected is usually not sparse, but in the transformation coefficient on an individual basis it is sparse or compressible.

The EMG is not sparse in the time domain, so it is necessary to design a suitable sparse transformation matrix to obtain the EMG signal projected onto the sparse matrix representation. Considering the embedded hardware system used in the wearable EMG monitoring device and the excellent performance of wavelets in signal compression [31], the sparse representation of EMG signals under a discrete wavelet transform (DWT) will be discussed. The Mallat decomposition of discrete sequences is performed for the myoelectric signal X. We construct a discrete wavelet transform matrix, i.e., the sparse matrix used in CS, as shown in Equations (3) and (4):

(3) $$G_{j+1}\left(n\right)=G\left(n\right)X_{j}\left(n\right)=\sum_{k=-\infty }^{\infty }G\left(k\right)X_{j}\left(n-k\right)$$

(4) $$H_{j+1}\left(n\right)=H\left(n\right)X_{j}\left(n\right)=\sum_{k=-\infty }^{\infty }H\left(k\right)X_{j}\left(n-k\right)$$

H(n) and G(n) are the high-pass and low-pass filter coefficient sequences corresponding to the selected wavelet function for both G(n) and H(n) ∈ $R^{l\times 1}$. When the decomposition level j=0, $X_{0}\left(n\right)$ is the EMG signal X to be compressed. After convolution, $G_{1}\left(n\right)$ $\in R^{\left(N+L-1\right)\times N}$. We remove the front L2−1 line and the rear L2−1 line and select even rows from the remaining N+1 rows, giving a total of N∕2 rows. Similarly, we select N∕2 rows from $H_{1}\left(n\right)$ and superimpose them to form a decomposed wavelet transform matrix $\Psi \in R_{N\times N}$. The N∕2 rows selected from $H_{1}\left(n\right)$ form the low-frequency part $X_{1}\left(n\right)$. The multi-layer decomposed wavelet transform matrix can be gained by continuing the iteration process according to Formulas (3) and (4).

A sparse basis needs to be exploited for sparse decomposition in compression measurements. To acquire a sparse basis suitable for EMG signals, a total of 52 types of wavelet bases in 6 wavelet functions are tested in this paper. They are haar, dbn, symn, coifn, bior, and rbio:

(5) x=ψθ

The transformation coefficient θ is sparse, and a few coefficients contain almost all of the energy. Combining Equations (2) and (5), the relationship between the perceptual data and transform coefficients can be expressed as follows:

(6) y=ϕx=ϕψθ 

If A (measurement matrix) = ϕψ, then:

(7) y=Aθ 

Since Equations (2) and (7) have the same form and assumption, the transformation coefficient can also be estimated according to the optimization method. Then, the signal x to be collected can be estimated through transformation. The sparsity of *x* itself can be regarded as the sparsity of x on the unit matrix, and the measurement matrix is the perception matrix.

When matrix A satisfies RIP, sparse signals can be recovered by minimizing the l1 norm. The more uncorrelated the perception matrix (ϕ) and the transformation matrix (ψ), the less sparse the signal that can be recovered [32]. Moreover, the random matrix’s probability that it is not related to any fixed transformation matrix is exceptionally high; that is to say, it is optimal to collect the unknown signal by collecting the random projection coefficient of the signal. In addition, the sparser the signal, the less perceptual data are needed to rebuild the original signal.

The compressed sensing method aims to restore all contents of the signal x through the measure y, which is far less than the amount of collected signal data.

There are two conditions under which recovery is possible [33]. Firstly, the signal is required to be sparse in a specific domain. The second one is incoherence, which is applied through the isometric property, which is sufficient for sparse signals [34].

Solving x from Formula (8) is an underdetermined problem, but on the other hand, the signal has only *K* unknown variables at unknown positions; that is, the signal has only *K* + 1 degrees of freedom. Therefore, under certain conditions, when the number of measurements exceeds the signal degrees of freedom, it can be recovered using some non-linear methods.

Obviously, when the *K* + 1 column of the perception matrix ϕ is selected arbitrarily and the linearity is independent, the signal with the sparsest characteristic found in all cases satisfying y=ϕx is required, solving the following optimization problem:

(8) $$min\|x{\|}_{0}\;\mathrm{subject}\;\mathrm{to}\;y=\phi x\;$$

where $\|x{\|}_{0}$ represents the zero norm of x; that is, the number of non-zero elements.

For the CS reconstruction, some greedy algorithms are proposed to reduce the amount of computation. The greedy algorithms include the matching pursuit [35], compressive sampling matching pursuit (CoSaMP) [36], iteratively reweighted least squares (Irls) [37], subspace pursuit (SP) [38], and stage-wise orthogonal matching pursuit [39] algorithms.

The OMP algorithm is a representative greedy algorithm. As shown in Algorithm 1, its principle is to add the best column of each iteration to the estimation. Then, to achieve orthogonality between the estimated value and the residual, the optimization operation is implemented and the least square method is used in the subspace of the selected best fitting columns [40].
**Algorithm 1** Orthogonal matching pursuit (OMP)**Input:** matrix ϕ, measurements *y*, sparsity *K***Output:** sparse reconstruction $x^{K}$1: $r^{0}=y$ and $\Gamma^{0}=\emptyset$
2: **for**  i=1…, *K* **do**3: $\lambda^{i}⇐argmax_{j}\left|\langle r^{i-1},\phi_{j}\rangle \right|$

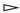
 Find best fitting column4: $\Gamma^{0}⇐\;\Gamma^{i-1}\cup^{\;}\;\lambda^{i}$
5: $x^{i}⇐argmin_{x}{\left||r^{i-1}-\phi_{\Gamma^{i}}x|\right|}_{2}^{2}$

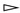
 LS optimization6: $r^{i}⇐$ $r^{i-1}-\phi_{\Gamma^{i}}x^{i}$

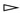
 Residual update7: **end for**

In the fifth step of Algorithm 1, the least squares (LS) optimization problem will be generated. QR decomposition (QRD) is applied to solve this problem. This process will be decomposed into two matrices, the unitary matrix and upper triangular matrix, respectively represented by Q and R. In the last step of the OMP iteration, a new iterative decomposition is reused via QRD to calculate Q and R.

However,$\;l_{0}$ is an NP hard problem [41], which can replace non-convex problems via the solving convex. As a classical convex optimization method, the BP algorithm is often employed for CS reconstruction. The L1 minimum norm is equivalent to the L0 minimum norm under certain conditions, and the same solution can be obtained. Then, the above Equation (8) can be transformed into the optimization problem under the minimum norm of L1, as shown in Equation (9):

(9) $$min\|x{\|}_{1}\;\mathrm{subject}\;\mathrm{to}\;y=\phi x$$

Under the condition of a restricted isometry property (RIP) [42], the minimum value of $\;l_{0}$ is equal to $\;l_{1}$, and $\;l_{1}$ can be found in polynomial time.

The theory of compressed sensing requires that the sensing matrix satisfy the RIP. For any k-sparse vector θ, if $\delta_{k}\in$(0,1) is satisfied then:

(10) $$\left(1-\delta_{k}\right){\left\|\theta \right\|}_{2}\leq {\left\|A\theta \right\|}_{2}\leq \left(1+\delta_{k}\right){\left\|\theta \right\|}_{2}\;$$

Then, matrix A satisfies the RIP.

However, it is arduous to verify the RIP conditions. If confirmed, cross-correlation is demonstrated as the equivalent condition of the RIP conditions. The measurement matrix ϕ is uncorrelated with the sparse representation matrix ψ; that is, any one of ϕ’s rows cannot be represented linearly by ψ’s columns, nor can any column of ψ be represented linearly by ϕ’s rows. Specifically, the correlation between the measurement matrix ϕ and sparse representation matrix ψ is defined as:

(11) $$\mu \left(\phi ,\psi \right)=\max\left|\langle \phi_{i},\psi_{j}\rangle \right|=\max\left|\langle A\left(i,j\right)\rangle \right|=\max\frac{\left|\langle a_{i},a_{j}\rangle \right|}{{\left\|a_{i}\right\|}_{2}{\left\|a_{j}\right\|}_{2}}\;1\leq i\leq j\leq N\;$$

In Formula (11), *ϕ* represents the measurement matrix and *ψ* is the sparse base. The correlation of ϕ and ψ is expressed by μ(ϕ,ψ). Here, $\phi_{i}$ and $\psi_{j}$ are row and column vectors of ϕ and ψ, respectively. In addition, $a_{i}$ and $a_{j}$ represents the ith row and jth column of matrix A, respectively. The more uncorrelated the matrix ϕ and sparse matrix ψ, the smaller the value of μ (ϕ, ψ), indicating the smaller the observation value that is required.

This paper is based on the existing compressed sensing theory for research. Although our theoretical innovations are limited, this ingenious sampling method is used to discuss the sparse basis and reconstruction algorithm of EMG signals in compressed sampling. Through the use of different compression ratios, a fixed measurement matrix, and a large number of calculations, the compressed sensing sparse basis and reconstruction algorithm most suitable for EMG signal are obtained.

### 2.3. Description of the EMG Datasets

A variety of physiological signals of the human body are stored in the MIT-BIH Polysomnographic Database [43], from which the EMG signals are extracted to analyze the compressed sensing accurately. We select the data for 5 channels here (slp32, slp37, slp41, slp45, slp48) and extract 100 fragments from the data for each channel, with 1024 points in each fragment. The sampling frequency is 250 Hz.

### 2.4. Performance Indicator

Several performance indicators are applied in this paper: the compression ratio (CR), percentage root mean square difference, and the time consumption.

### 2.5. Compression Ratio

Here, CR represents the measurement of the number of actual acquisition points required to collect the original signal x, which stands for the ratio of the number of actually collected signals to the number of original signals, along with the number of measurement M needed to obtain the accurately reconstructed signal. If M represents the dimensions of the measurement matrix and N represents the real length of the original signal x, CR can be expressed as:

(12) $$CR=1-\frac{M}{N}\;$$

Since *M* is less than *N*, the value of CR is less than 1. Therefore, in actual measurements, only a small amount of data is needed to obtain a large amount of plain text information. In this paper, a total of 9 CR values are selected from 0.1 to 0.9 at equal intervals.

### 2.6. Percentage Root Mean Square Distortion

The percentage root mean square distortion (PRD) is an indicator of the difference between the reconstructed signal $\hat{X}$ and the original signal X, defined as:

(13) $$PRD=\frac{{\left\|\hat{X}-X\right\|}_{2}}{{\left\|X\right\|}_{2}}\cdot 100$$

Lower PRD numbers represent better reconstruction performance. Another indicator is the execution time, reflecting the reconstruction algorithm’s efficiency.

### 2.7. Sparse Basis

Since EMG signals are not sparse in the time domain, a sparse basis needs to be utilized for sparse decomposition in the compression measurement. To acquire the sparse basis suitable for EMG signals, a total of 52 types of wavelet bases in 6 wavelet functions were tested in this paper. They were haar, dbn, symn, coifn, bior, and rbio, respectively.

### 2.8. Reconstruction Algorithms

The quality and efficiency of five kinds of commonly utilized reconstruction algorithms were tested to recover the signal. The five reconstruction algorithms were OMP, BP, CoSaMP, Irls, and SP.

### 2.9. Hardware Platform

The experiment was run on a laptop with a 1.9 GHz Intel Core i7-8650u CPU, 16 GB of memory, and a 512 GB hard disk. The program was run on MATLAB 2018b.

## 3. Results

This section may be divided by subheadings. It should provide a concise and precise description of the experimental results, their interpretation, as well as the experimental conclusions that can be drawn.

### 3.1. Compressed Sensing with Different Kinds of Wavelet Bases

Fifty-two different kinds of wavelet bases were applied for the sparse decomposition of EMG signals. The Bernoulli matrix and BP algorithm belonging to the convex optimization were exploited as fixed measurement matrices and reconstruction algorithms, respectively. Table 1 shows that the test effect, CR, and PRD values are inversely proportional. This means that the reconstruction quality is positively related to the number of measurements.

### 3.2. Compressed Sensing with Different Kinds of Construction Algorithms

In this paper, we applied the PRD and execution time as performance indicators to evaluate five kinds of reconstruction algorithms. In this test, the Bernoulli matrix and coif5 wavelet basis were employed as the fixed measurement matrix and sparse basis, respectively.

**Figure 3:**
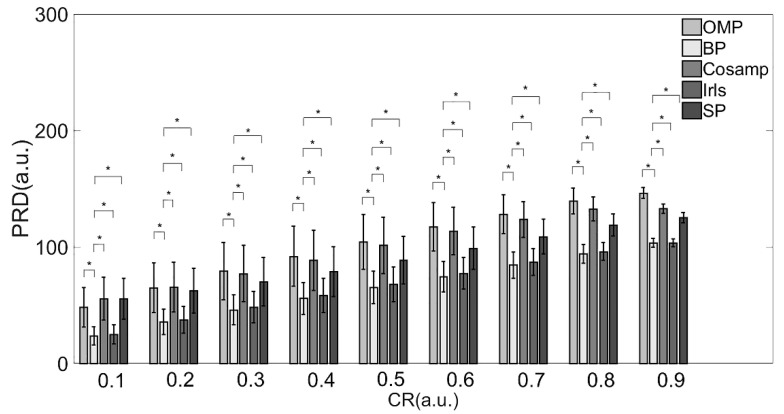
PRD values of various reconstruction algorithms. Note: * Indicates statistical significance via two-sample *t*-tests.

From the perspective of the deviation between the original and reconstructed signals shown in Table 2 and Figure 3, the BP and Irls reconstruction algorithms were better than the other three algorithms. To be more precise, the BP reconstruction algorithm has a better recovery effect under all compression ratios.

**Figure 4:**
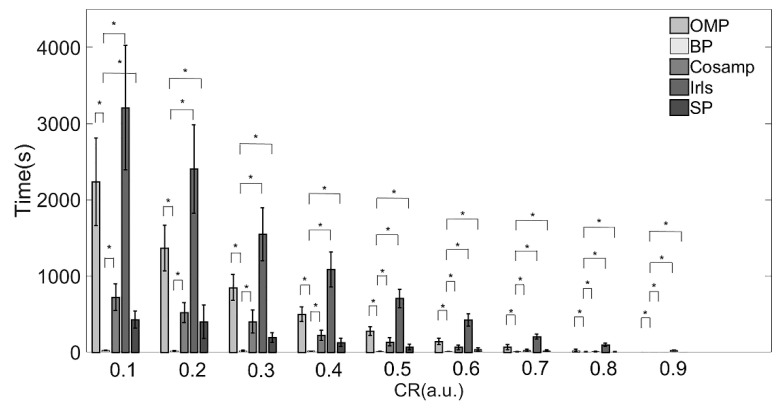
Execution times of various reconstruction algorithms. Note: * Indicates statistical significance via two-sample *t*-tests.

The execution times of the five reconstruction algorithms are shown in Table 3 and Figure 4. There were significant differences among each algorithm.

The IRLS algorithm consumed the most time under all compression ratios as compared with the other algorithms. When the CR increases from 0.1 to 0.9, the time consumed increases by more than 100 times. The reconstruction speed of the BP algorithm was the fastest under almost all compression ratios. When CR > 80%, the CoSaMP and SP algorithms took about the same time as the BP algorithm.

Combined with the results in Table 2 and Table 3 and Figure 3 and Figure 4, providing a comprehensive evaluation of the reconstruction quality and execution time, the BP reconstruction algorithm had superior reconstruction quality and high efficiency at all compression ratios. Although the Irls algorithm also showed high reconstruction quality, the execution efficiency was not satisfied. Conversely, the although CoSaMP and SP algorithms showed excellent efficiency when the CR was over 0.8, the reconstruction quality was poor. Therefore, considering these two factors, the BP algorithm was the most suitable for EMG signals in practical applications.

## 4. Discussion

The continuous and real-time monitoring of physiological signals play a significant role in disease diagnosis. However, in the long-term signal monitoring and wireless transmission of physiological signals, a large number of node resources will be required. Although the storage requirements can be satisfied due to Moore’s law, the physiological signal collection systems are also supposed to ensure the energy consumption efficiency. As an effective post-acquisition processing method, CS can reduce the power consumption and improve the physiological signal sampling efficiency significantly.

The experimental results indicated that via CS one can transmit and compress EMG signals efficiently. The db2 wavelet base was the most suitable sparse basis for EMG signals under various compression ratios and can be utilized as a sparse transform basis for EMG. In addition, under most compression ratios, the BP algorithm provided better reconstruction quality and higher reconstruction efficiency.

However, although the most suitable wavelet basis and reconstruction algorithm was acquired for EMG compressed sensing, there was still a gap between the rebuilt signal and the original one according to the PRD performance parameter. This implied that the existing convex optimization and greedy reconstruction algorithms cannot reorganize the information from the EMG signals well. A similar conclusion was also presented in [44]. Casson et al. used the BP reconstruction algorithm and cubic B-spline dictionary sparse basis to conduct a compression sensing sampling test on EMG signals, which also showed that the EMG signals could not be restored satisfactorily (the PRD values were above 50% in all cases). In our study, although the results were not very satisfactory, compared with the conclusions of Casson et al., when a suitable compression ratio is selected, the PRD value will be reduced to less than 50%. In practical applications, the db2 and BP algorithms can be used as the sparse basis and reconstruction algorithm, respectively, for the compression sensing sampling of EMG signals, and EMG signals with minor errors can be obtained by reducing the compression ratio.

In other words, the CS approach is not fully functional at present in terms of the post-acquisition processing. Nevertheless, the wavelet basis most suitable for compressed EMG sensing was found through a calculation and analysis process. Our future work will focus on EMG rebuilding process and deep learning applications in reconstruction.

## 5. Conclusions

This research aimed to evaluate the wavelet bases and reconstruction algorithms best suited for the compressive sampling of EMG signals. From the analysis based on the sampling quality and speed, it was concluded that the db2 wavelet basis provides a more balanced performance, while the BP reconstruction algorithm can achieve high accuracy in a short time.

## Figures and Tables

**Table 1:** PRD of reconstructed signals based on different wavelet bases. Values are means ± SD.

Wavelet	CR								
	10%	20%	30%	40%	50%	60%	70%	80%	90%
haar	30.67 ± 9.64	44.71 ± 15.35	56.62 ± 19.77	67.72 ± 21.97	78.64 ± 21.76	89.84 ± 21.33	100.41 ± 20.97	111.34 ± 20.98	120.4 ± 21.71
db2	23.74 ± 7.82	35.66 ± 10.63	45.99 ± 12.71	55.71 ± 13.83	65.23 ± 13.93	74.68 ± 13.09	84.34 ± 11.21	94.2 ± 7.93	103.48 ± 3.77
db3	34 ± 13.6	45.17 ± 15.65	55.86 ± 18.73	66.26 ± 20.84	77.4 ± 20.99	88.59 ± 19.82	99.2 ± 18.65	109.18 ± 17.97	115.23 ± 14.19
db4	24.15 ± 7.61	36.33 ± 10.45	46.9 ± 12.67	56.74 ± 13.94	66.32 ± 13.87	75.88 ± 12.83	85.49 ± 10.6	95.25 ± 7.36	103.65 ± 3.57
db5	34.51 ± 13.95	44.91 ± 14.47	54.38 ± 16.07	63.69 ± 16.75	73.42 ± 16.39	83.56 ± 14.94	93.56 ± 13.43	104.01 ± 11.12	112.27 ± 9.95
db6	26.25 ± 5.08	39.05 ± 6.1	50.01 ± 6.96	60.13 ± 7.2	69.63 ± 7.34	78.71 ± 6.83	87.81 ± 5.66	96.87 ± 3.88	104.78 ± 1.8
db7	26.4 ± 4.98	39.31 ± 5.96	50.19 ± 6.92	60.12 ± 7.26	69.65 ± 7	78.77 ± 6.6	87.91 ± 5.4	96.76 ± 3.74	104.87 ± 1.59
db8	26.5 ± 5.15	39.23 ± 6.17	50.09 ± 6.9	60.22 ± 7.02	69.8 ± 6.9	78.99 ± 6.15	88.17 ± 5.45	96.97 ± 3.83	105.06 ± 1.38
db9	26.55 ± 4.99	39.41 ± 6	50.43 ± 6.77	60.41 ± 7.09	69.92 ± 6.84	79.2 ± 6.34	88.16 ± 5.32	96.98 ± 3.71	104.93 ± 1.49
db10	26.65 ± 5.08	39.52 ± 6.05	50.42 ± 6.92	60.55 ± 6.97	69.82 ± 6.88	79.18 ± 6.27	88.12 ± 5.57	97.13 ± 3.66	104.99 ± 1.51
sym2	25.7 ± 5.1	38.51 ± 6.02	49.29 ± 7.08	59.37 ± 7.65	68.93 ± 7.56	78.25 ± 7.06	87.46 ± 6.07	96.44 ± 4.25	104.82 ± 1.61
sym3	25.81 ± 5.31	38.49 ± 6.26	49.4 ± 7.19	59.53 ± 7.45	68.94 ± 7.54	78.17 ± 6.89	87.44 ± 6.05	96.35 ± 4.19	104.64 ± 1.82
sym4	25.87 ± 5.2	38.58 ± 6.31	49.38 ± 7.16	59.44 ± 7.48	68.92 ± 7.71	78.27 ± 6.9	87.46 ± 6.16	96.39 ± 4.49	104.54 ± 1.81
sym5	25.93 ± 5.16	38.61 ± 6.2	49.49 ± 7.1	59.41 ± 7.46	69.03 ± 7.55	78.2 ± 6.99	87.41 ± 5.92	96.44 ± 4.17	104.63 ± 1.86
sym6	25.93 ± 5.16	38.67 ± 6.21	49.56 ± 7.17	59.54 ± 7.44	69.04 ± 7.74	78.33 ± 6.83	87.57 ± 6.03	96.36 ± 4.53	104.61 ± 1.8
sym7	26.03 ± 5.32	38.79 ± 6.21	49.55 ± 7.03	59.68 ± 7.36	69 ± 7.58	78.42 ± 6.84	87.42 ± 6.05	96.53 ± 4.24	104.68 ± 1.71
sym8	25.99 ± 5.13	38.73 ± 6.14	49.64 ± 7.15	59.62 ± 7.38	69.07 ± 7.7	78.35 ± 6.79	87.63 ± 5.98	96.4 ± 4.47	104.67 ± 1.7
coif1	25.65 ± 5.12	38.39 ± 6.07	49.19 ± 7.01	59.22 ± 7.68	68.78 ± 7.61	78.06 ± 7.03	87.33 ± 5.92	96.49 ± 4.24	104.5 ± 1.77
coif2	25.83 ± 5.21	38.51 ± 6.23	49.25 ± 7.13	59.41 ± 7.49	68.9 ± 7.62	78.06 ± 7	87.35 ± 6.08	96.22 ± 4.35	104.54 ± 1.8
coif3	25.95 ± 5.14	38.82 ± 6.17	49.55 ± 7.07	59.63 ± 7.39	69.15 ± 7.3	78.34 ± 6.98	87.54 ± 5.61	96.71 ± 4.09	104.47 ± 1.94
coif4	26.01 ± 5.12	38.77 ± 6.18	49.54 ± 7.12	59.64 ± 7.42	69.16 ± 7.47	78.29 ± 6.77	87.53 ± 5.86	96.39 ± 4.32	104.69 ± 1.57
coif5	26.05 ± 5.1	38.89 ± 6.12	49.73 ± 6.89	59.84 ± 7.32	69.25 ± 7.17	78.59 ± 6.57	87.69 ± 5.56	96.57 ± 4.06	104.7 ± 1.64
bior1.1	25.38 ± 5.22	38.16 ± 6.3	48.93 ± 7.15	59.22 ± 7.62	68.59 ± 7.79	77.89 ± 7.23	87.16 ± 6.47	96.3 ± 4.47	104.3 ± 2.35
bior1.3	26.13 ± 5.24	39.08 ± 6.3	50.02 ± 7.15	60.3 ± 7.49	69.84 ± 7.63	79.06 ± 7.09	88.03 ± 6.27	97.12 ± 4.09	104.76 ± 2.07
bior1.5	26.74 ± 5.32	39.89 ± 6.35	50.99 ± 7.15	61.28 ± 7.4	70.8 ± 7.56	79.95 ± 6.96	88.81 ± 6.19	97.68 ± 3.98	105.06 ± 1.99
bior2.2	27.19 ± 5.32	40.77 ± 6.25	52.45 ± 7.26	63.25 ± 7.7	73.29 ± 7.6	83.16 ± 6.78	92.33 ± 5.58	101.39 ± 3.46	108.44 ± 1.31
bior2.4	27.01 ± 5.31	40.46 ± 6.25	51.98 ± 7.13	62.56 ± 7.57	72.54 ± 7.48	82.12 ± 6.77	91.22 ± 5.52	100.33 ± 3.73	107.45 ± 1.33
bior2.6	27.12 ± 5.33	40.6 ± 6.26	52.09 ± 7.05	62.61 ± 7.49	72.56 ± 7.4	82.17 ± 6.75	91.22 ± 5.54	100.28 ± 3.79	107.41 ± 1.44
bior2.8	27.28 ± 5.33	40.83 ± 6.25	52.32 ± 7.02	62.83 ± 7.45	72.76 ± 7.32	82.4 ± 6.71	91.43 ± 5.51	100.39 ± 3.81	107.48 ± 1.45
bior3.1	31.7 ± 5.32	47.76 ± 6.35	61.99 ± 7.2	75.45 ± 7.31	88.62 ± 6.63	101.05 ± 5.78	114.82 ± 5.26	128.26 ± 3.62	135.14 ± 1.27
bior3.3	30.81 ± 5.07	45.97 ± 6.38	59.09 ± 7.18	71.06 ± 7.51	82.48 ± 6.86	93.13 ± 6.18	103.41 ± 4.95	113.08 ± 2.79	118.76 ± 0.9
bior3.5	30.59 ± 5.12	45.37 ± 6.41	58.1 ± 7.21	69.95 ± 7.44	80.92 ± 6.9	91.26 ± 6.02	100.76 ± 4.87	110.2 ± 2.87	115.69 ± 0.84
bior3.7	30.52 ± 5.2	45.12 ± 6.41	57.72 ± 7.17	69.49 ± 7.41	80.36 ± 6.94	90.51 ± 6.07	99.96 ± 4.93	109.31 ± 2.89	115.01 ± 0.85
bior3.9	28.26 ± 5.08	41.86 ± 6.01	53.02 ± 7.04	63.32 ± 7.44	72.8 ± 7.2	81.82 ± 6.59	90.73 ± 5.4	99.52 ± 3.82	106.96 ± 2.08
bior4.4	27.66 ± 5.05	40.99 ± 5.95	52.06 ± 6.98	62.27 ± 7.29	71.89 ± 7.03	81.06 ± 6.43	90.17 ± 5.35	99.19 ± 3.77	106.92 ± 1.97
bior5.5	27.71 ± 5.07	41.03 ± 5.97	52.08 ± 6.95	62.32 ± 7.19	71.96 ± 6.89	81.11 ± 6.29	90.26 ± 5.25	99.28 ± 3.64	107.03 ± 1.92
bior6.8	27.83 ± 5.08	41.19 ± 6.01	52.25 ± 6.94	62.53 ± 7.13	72.17 ± 6.8	81.29 ± 6.2	90.45 ± 5.16	99.43 ± 3.53	107.15 ± 1.87
rbio1.1	51.95 ± 6.79	67.99 ± 5.93	78.33 ± 5.85	85.43 ± 5.45	91.67 ± 4.53	97 ± 4.04	102.15 ± 3.49	107.48 ± 3.11	110.34 ± 2.02
rbio1.3	35.01 ± 5.42	52.02 ± 5.99	64.7 ± 6.58	74.85 ± 6.3	83.53 ± 5.58	91.01 ± 4.74	98.25 ± 3.97	105.47 ± 3.28	110.4 ± 2.1
rbio1.5	32.77 ± 5.26	48.84 ± 6	61.35 ± 6.63	71.9 ± 6.64	81.07 ± 5.98	89.16 ± 4.99	96.97 ± 4.23	104.85 ± 3.29	110.48 ± 2.09
rbio2.2	32.44 ± 5.2	48.1 ± 6.06	60.4 ± 6.69	70.97 ± 6.71	80.14 ± 6.1	88.41 ± 5.18	96.51 ± 4.25	104.61 ± 3.34	110.57 ± 2.08
rbio2.4	32.4 ± 5.19	47.84 ± 6.13	60.06 ± 6.72	70.61 ± 6.73	79.78 ± 6.13	88.18 ± 5.24	96.38 ± 4.21	104.55 ± 3.35	110.63 ± 2.11
rbio2.6	26.28 ± 5.16	39.22 ± 6.13	50.06 ± 7.12	60.13 ± 7.62	69.82 ± 7.49	79.08 ± 6.87	88.16 ± 5.78	97.04 ± 4.05	104.8 ± 1.8
rbio2.8	26.57 ± 5.3	39.77 ± 6.33	50.96 ± 7.18	61.36 ± 7.69	71.29 ± 7.64	80.76 ± 7.05	89.85 ± 5.76	98.73 ± 4	106.28 ± 1.68
rbio3.1	26.19 ± 5.13	39.06 ± 6.13	49.83 ± 6.98	59.83 ± 7.46	69.44 ± 7.28	78.71 ± 6.7	87.85 ± 5.63	96.82 ± 3.94	104.92 ± 1.8
rbio3.3	28.26 ± 5.08	41.86 ± 6.01	53.02 ± 7.04	63.32 ± 7.44	72.8 ± 7.2	81.82 ± 6.59	90.73 ± 5.4	99.52 ± 3.82	106.96 ± 2.08
rbio3.5	27.66 ± 5.05	40.99 ± 5.95	52.06 ± 6.98	62.27 ± 7.29	71.89 ± 7.03	81.06 ± 6.43	90.17 ± 5.35	99.19 ± 3.77	106.92 ± 1.97
rbio3.7	27.71 ± 5.07	41.03 ± 5.97	52.08 ± 6.95	62.32 ± 7.19	71.96 ± 6.89	81.11 ± 6.29	90.26 ± 5.25	99.28 ± 3.64	107.03 ± 1.92
rbio3.9	27.83 ± 5.08	41.19 ± 6.01	52.25 ± 6.94	62.53 ± 7.13	72.17 ± 6.8	81.29 ± 6.2	90.45 ± 5.16	99.43 ± 3.53	107.15 ± 1.87
rbio4.4	51.95 ± 6.79	67.99 ± 5.93	78.33 ± 5.85	85.43 ± 5.45	91.67 ± 4.53	97 ± 4.04	102.15 ± 3.49	107.48 ± 3.11	110.34 ± 2.02
rbio5.5	35.01 ± 5.42	52.02 ± 5.99	64.7 ± 6.58	74.85 ± 6.3	83.53 ± 5.58	91.01 ± 4.74	98.25 ± 3.97	105.47 ± 3.28	110.4 ± 2.1
rbio6.8	32.77 ± 5.26	48.84 ± 6	61.35 ± 6.63	71.9 ± 6.64	81.07 ± 5.98	89.16 ± 4.99	96.97 ± 4.23	104.85 ± 3.29	110.48 ± 2.09

**Table 2:** PRD values of the reconstruction signals for the different compression ratios (CRs) based on the same wavelet basis. Values are means ± SD.

Method	CR								
	0.1	0.2	0.3	0.4	0.5	0.6	0.7	0.8	0.9
OMP	48.24 ± 16.85	65.04 ± 21.35	79.3 ± 24.66	92.13 ± 25.64	104.56 ± 23.64	117.41 ± 20.89	128.26 ± 16.89	139.69 ± 11.11	146.37 ± 4.82
BP	23.87 ± 7.98	35.71 ± 10.75	46.1 ± 12.81	55.87 ± 13.86	65.31 ± 13.97	74.71 ± 13.07	84.48 ± 11.31	94.23 ± 7.95	103.51 ± 3.86
CoSaMP	55.61 ± 18.29	65.58 ± 21.42	77.07 ± 24.22	88.58 ± 25.93	101.44 ± 24.29	113.75 ± 20.46	123.59 ± 15.49	132.66 ± 10.19	132.89 ± 4.04
Irls	24.99 ± 8.44	37.53 ± 11.39	48.37 ± 13.65	58.42 ± 14.87	68.03 ± 14.85	77.49 ± 13.62	87 ± 11.47	96.1 ± 7.79	103.63 ± 3.59
SP	55.51 ± 17.55	62.37 ± 19.22	70.17 ± 20.76	78.89 ± 21.43	88.76 ± 20.44	98.88 ± 18.09	108.94 ± 14.9	119.03 ± 9.39	125.35 ± 4.43

**Table 3:** Execution times (in seconds) of various reconstruction algorithms. Values are mean ± SD.

Method	CR								
	0.1	0.2	0.3	0.4	0.5	0.6	0.7	0.8	0.9
OMP	2236.66 ± 574.21	1368.17 ± 300.14	849.29 ± 169.05	496.98 ± 96.43	275.77 ± 57.91	141.54 ± 41.09	65.38 ± 32.34	23.88 ± 17.25	21.01 ± 0.82
BP	24.35 ± 3.71	20.49 ± 3.18	19.55 ± 6.75	13.43 ± 1.62	10.68 ± 1.75	9 ± 2.73	6.8 ± 2	5.43 ± 1.51	3.89 ± 0.82
CoSaMP	722.35 ± 173.09	519.86 ± 130.66	401.89 ± 152.8	221.14 ± 64.39	134.06 ± 54.4	63.89 ± 29.86	26.77 ± 15.09	9.69 ± 5.96	1.9 ± 1.4
Irls	3207.79 ± 817.23	2405.05 ± 578.46	1548.56 ± 346.9	1086.58 ± 230.09	705.22 ± 120.33	423.7 ± 83.59	206.53 ± 34.87	94.26 ± 20.81	26.17 ± 9.02
SP	431.65 ± 112.13	397.19 ± 221.28	194.22 ± 61.99	128.29 ± 55.02	68.97 ± 35.96	36.75 ± 20.47	18.9 ± 12.11	4.48 ± 1.7	1.33 ± 0.87

## Data Availability

Not applicable.
